# Cervical cancer with BRCA1 gene mutations: case reports and literature review

**DOI:** 10.3389/fonc.2026.1742990

**Published:** 2026-04-22

**Authors:** Jian Yang, Liang Song, Rutie Yin, Lan Zhong

**Affiliations:** 1 Department of Obstetrics and Gynecology, West China Second University Hospital, Sichuan University, Chengdu, Sichuan, China; 2 Key Laboratory of Birth Defects and Related Diseases of Women and Children, Sichuan University, Ministry of Education, Chengdu, Sichuan, China

**Keywords:** BRCA gene mutation, cervical cancer, management, prognosis, treatment strategy

## Abstract

**Background:**

Mutations in breast cancer susceptibility genes (BRCA1 and BRCA2) are well-established risk factors for breast, ovarian, prostate, and pancreatic cancers. However, their occurrence in cervical cancer is rare, with a reported prevalence of less than 5%. To date, only a limited number of cervical cancer cases harboring BRCA mutations have been reported.

**Case presentation:**

We conducted a retrospective analysis of two cases of cervical cancer with germline BRCA1 mutations. Case 1: A 39-year-old woman presented with poorly differentiated squamous cell carcinoma of the cervix, classified as International Federation of Gynecology and Obstetrics (FIGO) stage IIA1. After radical hysterectomy, she received adjuvant chemoradiotherapy. Owing to a family history of cancer, genetic testing was performed, revealing a pathogenic germline BRCA1 mutation. One year later, she underwent prophylactic bilateral salpingo-oophorectomy. After 44 months of rigorous follow-up, no evidence of disease recurrence was observed. Case 2: A 26-year-old woman was diagnosed with poorly differentiated squamous cell carcinoma of the cervix, FIGO 2018 stage IVB. She received neoadjuvant chemotherapy followed by concurrent chemoradiotherapy and four additional cycles of combination chemotherapy. Whole-exome sequencing identified a germline BRCA1 mutation. Two years after the completion of initial treatment, imaging revealed metastatic involvement of lymph nodes in the left axilla. She subsequently received six cycles of palliative chemotherapy. At 50 months post-diagnosis, the patient remains alive under close clinical surveillance.

**Conclusion:**

In patients with cervical cancer, BRCA testing holds important clinical value, particularly for the management of those with a significant family history of malignancy or confirmed hereditary predisposition. Appropriate genetic counseling plays an essential role in guiding preventive strategies, facilitating early diagnosis, and supporting informed decision-making regarding potential prophylactic interventions.

## Background

Throughout their lifespan, cells are continuously exposed to various forms of genomic damage. To maintain genomic integrity, cells rely on highly regulated mechanisms to detect and repair DNA lesions (1). Among these, the breast cancer susceptibility genes (BRCA1 and BRCA2) encode key tumor suppressor proteins that are essential for the repair of DNA double-stranded breaks through homologous recombination. Pathogenic mutations in BRCA1 and BRCA2 are associated with significantly increased risk of hereditary malignancies, primarily breast and ovarian cancer, and have additionally been implicated in pancreatic, prostate, colorectal, and cervical cancers (2–4). However, reports of co-occurrence of cervical cancer with BRCA gene mutations remain extremely rare, posing unique challenges in the diagnosis, treatment, and long-term clinical management of BRCA-related cervical cancer. Here, we report two cases of cervical cancer with BRCA mutations, with the aim of expanding the existing clinical evidence for this rare condition.

## Case presentation

### Case 1

**Figure 1:**
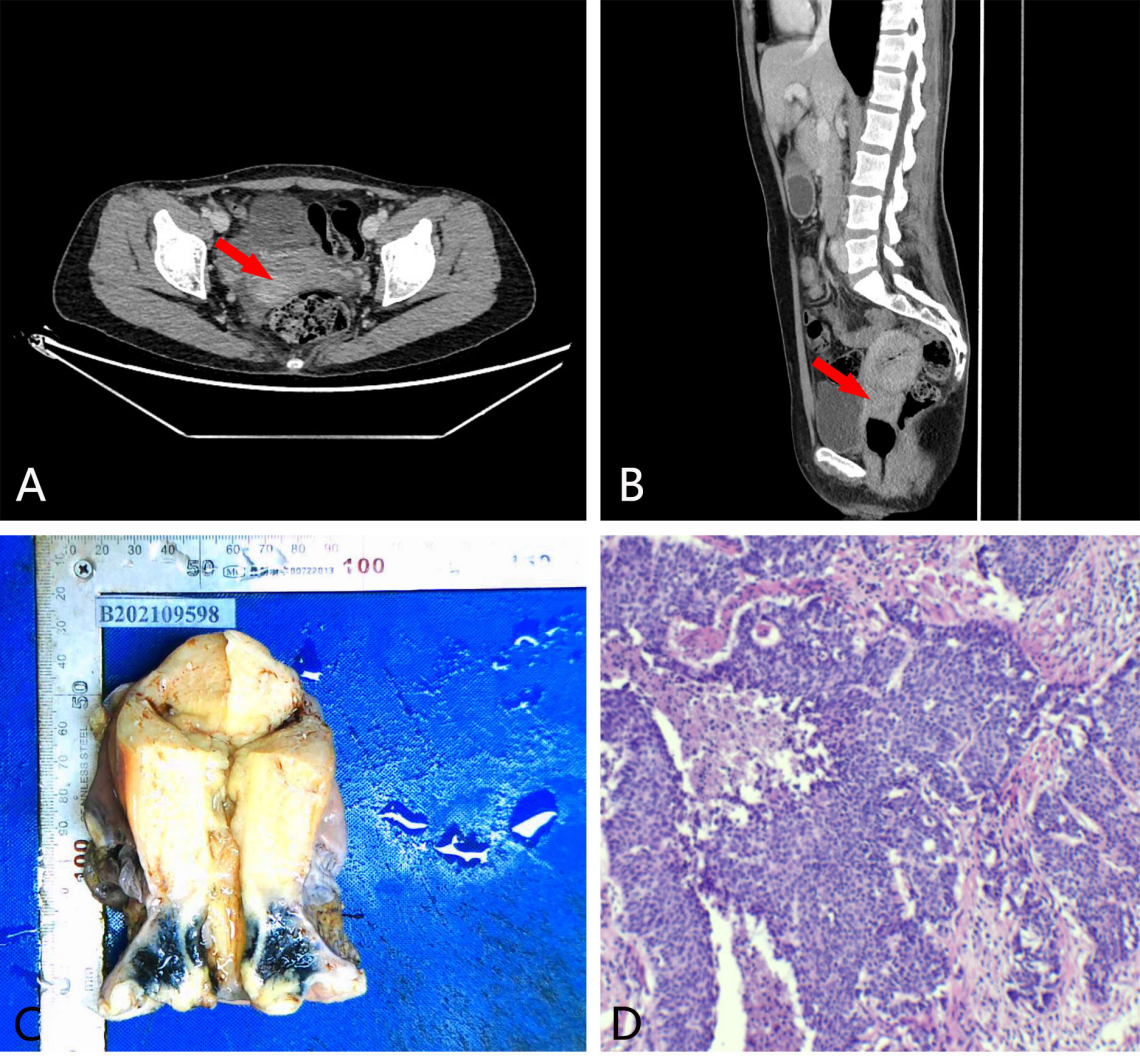
Imaging and surgical specimens of case 1. **(A, B)** cervical mass. **(C)** the gross specimen after the operation. **(D)** the microscopic specimen after the operation (10×).

In May 2021, a 39-year-old woman presented to West China Second University Hospital, Sichuan University, with a history of post-coital vaginal bleeding for several months. Gynecological examination revealed a 4 cm exophytic ulcerative lesion of the cervix, extending into the vaginal fornices. The bilateral uterosacral ligaments were soft and elastic. Cervical biopsy confirmed poorly differentiated squamous cell carcinoma (SCC) with concurrent human papilloma virus (HPV) 18 infection. Computed tomography (CT) showed a cervical mass extending into the upper vagina, with no evidence of enlarged lymph nodes or distant metastasis. According to the International Federation of Gynecology and Obstetrics (FIGO) 2018 staging system, the patient was diagnosed with stage IIA1 poorly differentiated SCC of the cervix. She underwent extensive total hysterectomy pelvic lymphadenectomy, and para-aortic lymph node sampling with ovarian preservation. Pathological examination revealed tumor invasion into the deep one-third (>1/2) of the cervical stroma, intravascular tumor thrombus, and involvement of the vaginal vault and surgical margin, but no extension to the parametria or pelvic sidewalls. A total of 32 pelvic and 2 para-aortic lymph nodes were examined, all of which were negative for metastatic disease. Given the presence of high-risk pathological features, the patient received adjuvant cisplatin-based chemoradiotherapy as follows: 50.4 Gy to the pelvis (with dose escalation to 56.0 Gy at the vaginal stump and 61.6 Gy to involved lymph node areas), two brachytherapy sessions, and five cycles of concurrent cisplatin chemotherapy. Due to a family history of malignancy, genetic testing was performed, which led to the identification of a germline BRCA1 mutation (BRCA1 p.K1208Nfs*2). This finding was associated with a substantially elevated risk of ovarian cancer. To reduce this risk, the patient underwent preventive bilateral salpingo-oophorectomy one year after the completion of primary treatment. Upon postoperative histopathological examination, no tumor cells were detected. After 44 months of close follow-up, the patient remained disease-free with no evidence of tumor recurrence (**Figure 1**).

### Case 2

**Figure 2:**
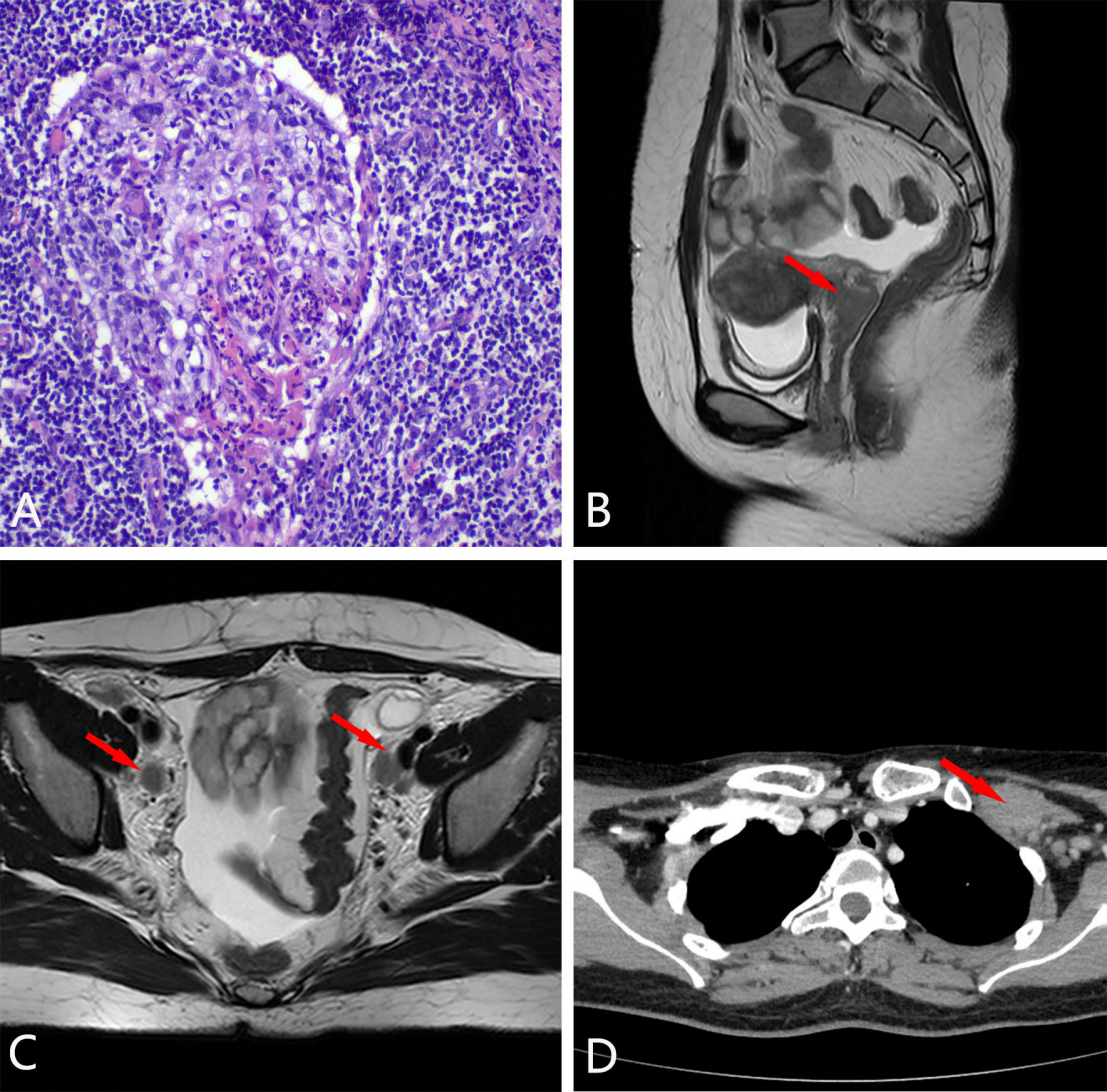
The pathology and imaging of case 2. **(A)** the biopsy pathological specimen (10×). **(B)** cervical lesion. **(C)** enlarged lymph nodes in the pelvic area. **(D)** enlarged lymph nodes on the left axilla.

In September 2021, a 26-year-old woman presented to our hospital with a six-month history of postcoital vaginal bleeding. Gynecological examination revealed a 2 cm cauliflower-like cervical lesion involving the upper two-thirds of the vagina. The uterosacral ligaments were normal. Cervical biopsy confirmed poorly differentiated squamous cell carcinoma with positivity for human papillomavirus type 16 (HPV16). Abdominal and pelvic magnetic resonance imaging (MRI) scans revealed a cervical mass with multiple enlarged lymph nodes in the para-aortic, common iliac, and obturator regions. A subsequent 18F-FDG positron emission tomography (PET) scan confirmed the presence of a cervical mass with distant nodal metastases involving the left supraclavicular, right diaphragmatic foot area, retroperitoneal large blood vessels, bilateral common iliac vessels, right iliac fossa, and bilateral pelvic walls. No clear evidence of other systemic spread was observed. Based on the FIGO 2018 staging system, the patient was diagnosed with stage IVB cervical cancer. She commenced neoadjuvant chemotherapy consisting of paclitaxel, cisplatin, and bevacizumab for two cycles. Genetic testing was conducted in view of her family history of malignancy, which identified a germline BRCA1 mutation (c.4739_4743delCTGAA, p.S1580*). She subsequently received concurrent chemoradiotherapy as follows: 50.4 Gy to the pelvis, 61.6 Gy to involved lymph node areas, five brachytherapy sessions, and five cycles of cisplatin. This was followed by four cycles of adjuvant combination chemotherapy with paclitaxel and carboplatin. The patient achieved complete clinical remission and remained disease-free for two years until an imaging examination revealed metastatic involvement in the left axillary lymph nodes, for which she received an additional six cycles of palliative chemotherapy. At 50 months post-diagnosis, the patient remains alive under close monitoring (**Figure 2**).

### Ethical approval and informed consent

The appropriate informed consents were obtained and the study was approved by the Ethics Committee of West China Second University Hospital of Sichuan University (Sichuan, China) (Approval No.: 2020076).

## Discussion

Since the discovery of BRCA1 and BRCA2 in the 1990s, substantial progress has been made in understanding the natural history and risk management of women carrying pathogenic variants in these genes. As key tumor suppressors, BRCA1/2 proteins are essential for the repair of DNA double-strand breaks (via homologous recombination). Mutations in BRCA genes can cause genomic instability, thereby increasing the risk of multiple malignancies, including breast, ovarian, prostate, and pancreatic cancers. Earlier research demonstrates that in breast cancer cells lacking BRCA1, expression of HPV16 oncogenic proteins significantly enhances cell proliferation and invasive capacities, that indicating a possible synergistic effect between viral oncogenesis and impaired DNA repair mechanisms (5, 6). Consistently, Naciri et al. (2). reported an earlier onset of cervical cancer in women with BRCA1 mutations, suppporting a potential interaction between genetic predisposition and viral carcinogenesis. This observation is concordant with the two cases described in this study, in which cervical cancer was diagnosed at relatively young ages (39 and 26 years), both substantially earlier than the typical age of onset. The collective findings support the hypothesis that BRCA mutations contribute to accelerated tumorigenesis in genetically susceptible individuals. However, given the limited sample size, these findings should be interpreted with caution and large-scale studies are required to draw more precise conclusions.

To date, large-scale epidemiological studies have not demonstrated a significant increase in the overall risk of cervical cancer among individuals carrying pathogenic BRCA gene variants. In fact, only a limited number of individual cases of cervical cancer associated with BRCA gene mutations have been reported (**Table 1** summarizes the published cases of cervical cancer in patients with BRCA gene mutations). Feldman et al. (8) conducted an integrated analysis of 592 cervical cancer samples from a U.S. tumor repository combining next-generation sequencing, gene amplification assessment via fluorescence *in situ* hybridization (FISH), and protein expression profiling via immunohistochemistry. Their study identified BRCA1 gene mutations in 3.7% of cases, indicating that in the real world, the coexistence of cervical cancer and BRCA mutations may be more common than we originally thought. Currently, BRCA testing is not recommended as a routine component of clinical guidelines for cervical cancer. Consequently, the number of detected BRCA-mutated cervical cancer cases remains substantially lower than the estimated prevalence of BRCA carriers, leading to limited characterization of the genomic landscape of BRCA dysfunction in this patient population. This gap hinders the development of individualized surveillance and management strategies and precludes robust comparisons of clinical outcomes between cervical cancer patients with and without BRCA mutations. Despite recent reductions in sequencing costs and improvements in throughput, genetic testing remains expensive and time-consuming. Future advancements that further reduce costs and enhance accessibility and efficiency could enable broader implementation of comprehensive genomic profiling, facilitate personalized treatment approaches, and allow for accurate estimation of the true incidence of BRCA mutations in cervical cancer.

**Table 1:** The summary of BRCA gene mutations in cervical cancer in the existing literature.

Case number	Year reported	Age	Figo stage	Treatment strategy	PFS	OS	Outcome
1	2025 (2)	30	IIB	concurrent chemoradiotherapy	6 years	16 years	alive
2	2015 (3)	–	–	–	–	–	–
3	2021 (7)	49	IIIC2	concurrent chemoradiotherapy	36	36	alive

PFS, Progress Free Survival; OS, Overall Survival.

Notably, the occurrence of cervical cancer in individuals with BRCA mutations has raises important questions regarding the need for increased awareness of BRCA-related risks, particularly the predisposition to multiple primary malignancies and the importance of genetic counseling. For patients with BRCA mutations, healthcare providers should consider conducting routine surveillance for non-breast and non-ovarian malignancies, including cervical, pancreatic, and colorectal cancers. Personalized surveillance and preventive strategies may facilitate earlier detection of secondary malignancies, thereby improving clinical outcomes and overall survival rates. In young patients diagnosed with cervical cancer, consideration should be given to risk-reducing interventions, including prophylactic surgeries such as bilateral salpingo-oophorectomy, may be considered to mitigate the risk of subsequent ovarian cancer. Such decisions necessitate thorough counseling on the risks and benefits of preventive measures. Moreover, genetic counseling is essential not only for the patient but also for at-risk family members, who may benefit from cascade genetic testing and early cancer screening (2). For the management of such patients, tailoring therapeutic strategies according to the patient’s genetic profile and understanding the interplay among different malignancies in the context of BRCA mutations may be beneficial in improving the quality of life and survival rates of these complex cases.

To date, several studies have aimed to elucidate the association between BRCA expression and clinical prognosis in cervical cancer. However, the findings remain inconsistent. For instance, Narayan et al. (9) reported the presence of BRCA1 promoter hypermethylation is present in 6.1% of cervical cancer patients. Hypermethylation of the FANCF gene promoter may disrupt the Fanconi anemia-BRCA (FA-BRCA) pathway, leading to cisplatin resistance in cervical cancer. In contrast, other studies have demonstrated that BRCA-deficient cells exhibit impaired DNA damage repair via homologous recombination (HR) (10, 11), rendering them more sensitive to certain chemotherapeutic agents. Additionally, Balacescu et al. (12) found that overexpression of BRCA1 and BRCA2 in patients with advanced cervical cancer is associated with poorer treatment response. Similarly, a recent *in vitro* investigation by Wen et al. (13) further suggested that BRCA1 overexpression may contribute to increased resistance to chemoradiotherapy in cervical squamous cell carcinoma. These discrepancies may be attributed not only to the established relationship between gene alterations and cancer prognosis but also to differences in mRNA and protein expression levels, which can be influenced by post-transcriptional and post-translational regulatory mechanisms. Therefore, Further investigation is therefore warranted to clarify these complex molecular dynamics (4).

Elucidation of molecular alterations underlying cancer development has driven the emergence of targeted therapies and more personalized approaches to patient management. Although poly (ADP-ribose) polymerase (PARP) inhibitors represent an established therapeutic option for patients with BRCA mutations, they were not utilized in the present cases. Instead, we adhered to the standard treatment regimen recommended by current clinical guidelines. In the event of standard treatment failure, enrollment in clinical trials evaluating PARP inhibitors would be actively considered. Several small-scale case reports and early-phase clinical trials have already begun to explore the use of PARP inhibitors in cervical cancer, particularly in recurrent or refractory settings. Emerging evidence indicates that a subset of patients with recurrent or metastatic cervical cancer resistant to conventional chemotherapy, even in the absence of germline BRCA mutations, may harbor tumors with homologous recombination repair deficiency (HRD). In such cases, PARP inhibitors, administered either as monotherapy or in combination with chemotherapy, immunotherapy, or other agents, have demonstrated promising therapeutic activity, as highlighted in the CLAP study (14). These findings strongly support the expansion of predictive biomarkers for PARP inhibitor efficacy in cervical cancer beyond BRCA mutations to include a broader HRD status. To date, effective options for gynecologic cancer risk reduction remain limited. In the long term, advancements in screening methods, chemopreventive agents, and a mechanistic deeper understanding of the molecular pathogenesis of hereditary cervical cancer will be essential to eventually render prophylactic organ removal obsolete (15). The present study has a number of limitations that should be taken into consideration. The primary source of potential bias lies in the comprehensiveness of the literature review. Additionally, given the inherent limitations of analysis based on the review and the selective nature of reported cases in the literature, our findings may subjected to publication bias.

In conclusion, we report two rare cases of cervical cancer associated with BRCA mutations, providing detailed accounts of treatment strategies and prognostic insights, with the aim of contributing to the growing evidence base informing the clinical management of this rare condition. Pathogenic BRCA gene mutations confer multiple oncogenic risks and necessitate comprehensive, individualized, and multidisciplinary approaches to cancer management.

## Data Availability

The original contributions presented in the study are included in the article/supplementary material. Further inquiries can be directed to the corresponding author.
